# Generation of a sub-diffraction hollow ring by shaping an azimuthally polarized wave

**DOI:** 10.1038/srep37776

**Published:** 2016-11-23

**Authors:** Gang Chen, Zhi-xiang Wu, An-ping Yu, Zhi-hai Zhang, Zhong-quan Wen, Kun Zhang, Lu-ru Dai, Sen-lin Jiang, Yu-yan Li, Li Chen, Chang-tao Wang, Xian-gang Luo

**Affiliations:** 1 Key Laboratory of Optoelectronic Technology and Systems (Chongqing University), Ministry of Education, and Key Disciplines Lab of Novel Micro-nano Devices and System Technology, Chongqing University, 173 Shazheng Street, Shapingba, Chongqing 400044, China; 2 National Center for Nanoscience and Technology, No. 11 Zhong Guan CunBei Yi Tiao, Beijing 100190, China; 3 State Key Laboratory of Optical Technologies on Nano-Fabrication and Micro-Engineering, Institute of Optics and Electronics, Chinese Academy of Science, P. R. Box 350, Chengdu 610209, China

## Abstract

The generation of a sub-diffraction optical hollow ring is of great interest in various applications, such as optical microscopy, optical tweezers, and nanolithography. Azimuthally polarized light is a good candidate for creating an optical hollow ring structure. Various of methods have been proposed theoretically for generation of sub-wavelength hollow ring by focusing azimuthally polarized light, but without experimental demonstrations, especially for sub-diffraction focusing. Super-oscillation is a promising approach for shaping sub-diffraction optical focusing. In this paper, a planar sub-diffraction diffractive lens is proposed, which has an ultra-long focal length of 600 λ and small numerical aperture of 0.64. A sub-diffraction hollow ring is experimentally created by shaping an azimuthally polarized wave. The full-width-at-half-maximum of the hollow ring is 0.61 λ, which is smaller than the lens diffraction limit 0.78 λ, and the observed largest sidelobe intensity is only 10% of the peak intensity.

In recent years, there has been a growing interest in generating and focusing cylindrically polarized waves due to their unique properties and possible application in light shaping^1^. The hollow rings have great potential in many optical applications, such as optical microscopy^2^, optical tweezers^3^, and nanolithography^4^. Reducing hollow ring size is of particular importance in further enhancing the optical resolution. Hollow focal spots can be generated by utilizing nanostructures^5^ and the vortex phase plate^6^. An alternative way to generate a tight hollow ring is to focus an azimuthally polarized wave. Recently, special attention has been given to azimuthally polarized waves because of their unique properties in focusing and microscopy. Focusing of azimuthally polarized wave by high numerical aperture (NA) aplanatic lens has been extensively studied theoretically^6^,^7^,^8^,^9^,^10^,^11^,^12^,^13^,^14^. For a wavelength λ, a full width at half maximum (FWHM) of 0.24 λ of a ring size was theoretically predicted for an objective lens with a numerical aperture (NA) value of 1.4. However, its central ring peak was surrounded by a huge sidelobe ring, which was about 44 times larger than the central ring peak itself^14^. Planar focusing lenses are more attractive than conventional optical lenses because they are small, lightweight, and easily integrated. A planar plasmonic metalens based on the parabolic phase profile was proposed for the tight focusing of azimuthally polarized light, but the FWHM of the hollow ring was still larger than the diffraction limit^15^. Perfect lenses based on negative refraction have also been proposed for sub-diffraction focusing and imaging with resolution of a few nanometers for visible light^16^,^17^. However, like those lenses^18^,^19^,^20^ utilizing evanescent wave, their working distance is limited within a wavelength. Super-oscillation is an effective way in shaping sub-diffraction optical structure in far-field. To achieve a long working distance, super-oscillatory lenses have been proposed for far-field sub-wavelength focusing beyond the diffraction limit^21^,^22^,^23^,^24^,^25^,^26^,^27^,^28^. Recently, lenses based on quasi-continuous amplitude modulation^29^ and binary amplitude-phase planar mask^30^ have been demonstrated for the sub-diffraction focusing of linearly polarized light in experiments. A binary amplitude-phase-mask-based super-oscillation lens was also reported for the sub-diffraction focusing of circularly polarized waves with an ultra-long focal length and small NA^31^. Metamaterials^32^,^33^ are promising building block for super-resolution lens. A lens based on metasurface was reported for ultrabroadband sub-diffraction focusing^34^, however present metasurface suffers from the low transmission rate. Super-oscillartory lenses were also reported for applications, such as super-resolution microscopes^24^,^35^,^36^ and telescope^37^. This provides a new way to generate a sub-diffraction optical hollow ring. In this paper, we propose a far-field diffractive planar lens based on binary phase mask, which is designed with an ultra-long focal length of 600 λ and a small NA of 0.64. The lens focuses an azimuthally polarized wave into sub-diffraction hollow ring with an FWHM of 0.61 λ, smaller than the diffraction limit 0.78 λ. A small sidelobe level was also observed on the focal plane.

## Materials and Methods

### Theoretical design of sub-diffraction lens

**Figure 1:**
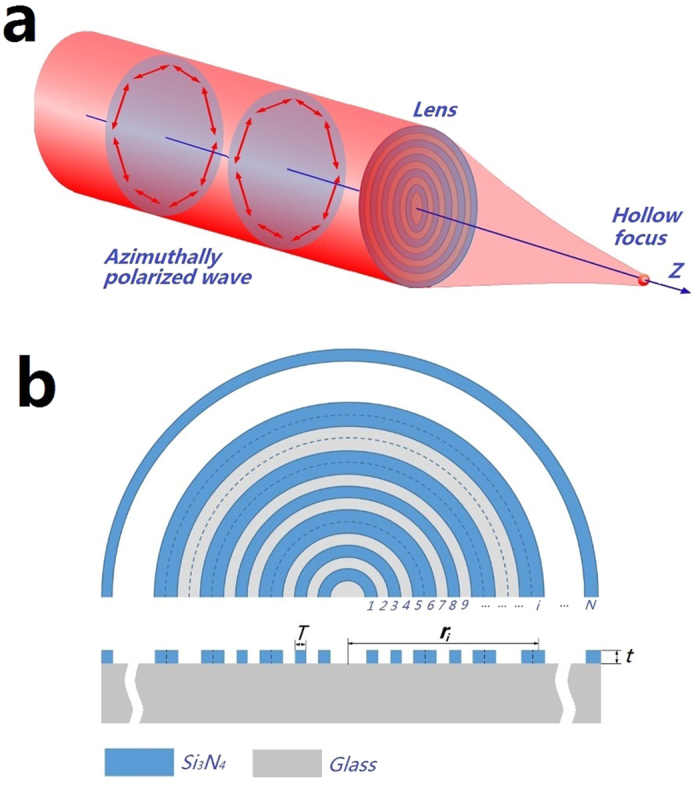
(**a**) Generation of a sub-diffraction hollow ring by shaping the azimuthally polarized wave with a planar binary phase lens, and (**b**) the micro lens structure.

Figure 1(a) illustrates the generation of the sub-diffraction hollow ring by shaping the azimuthally polarized wave with a planar binary phase lens. The geometrical structure of the lens is depicted in Fig. 1(b). The lens consists of a series of concentric Si_3_N_4_ rings grown on the top of a glass substrate. The width and the thickness of the rings are *T* and *t*, respectively. The binary phase was realized by controlling the Si_3_N_4_ stripe thickness 0 and *t* for the phase changes of 0 and π, respectively. The value of *t* was obtained by *t* = *λ*/2(*n*_Si3N4_-1), with *n*_Si3N4_ being the refractive index of Si_3_N_4_.

A lens based on binary phase modulation was designed for a normally incident wave with an azimuthal polarization at wavelength λ = 632.8 nm. In the design, the diffracted electrical field was calculated with angular spectrum method^21^, and the lens phase spatial distribution was optimized by using particle swarm optimization algorithm^38^. The electrical field of an azimuthally polarized wave can be expressed by the superposition of linearly polarized TEM_01_ and TEM_10_ Gaussian modes^39^, as given by Equation 1, where *E*_0_ is the incident electrical field amplitude, *w*_0_ is the beam waist size, *z*_0_ = *πw*_0_^*2*^/λ is the Rayleigh range, *R*(*z*) = *z*[1+(*z*_0_/*z*)^2^] is the radius of curvature, *w*(*z*) = *w*_0_[1+(*z*/*z*_0_)^2^]^1/2^ is the beam width at *z*, and k = 2 *π/*λ is the wavenumber.


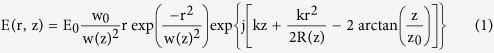


**Figure 2:**
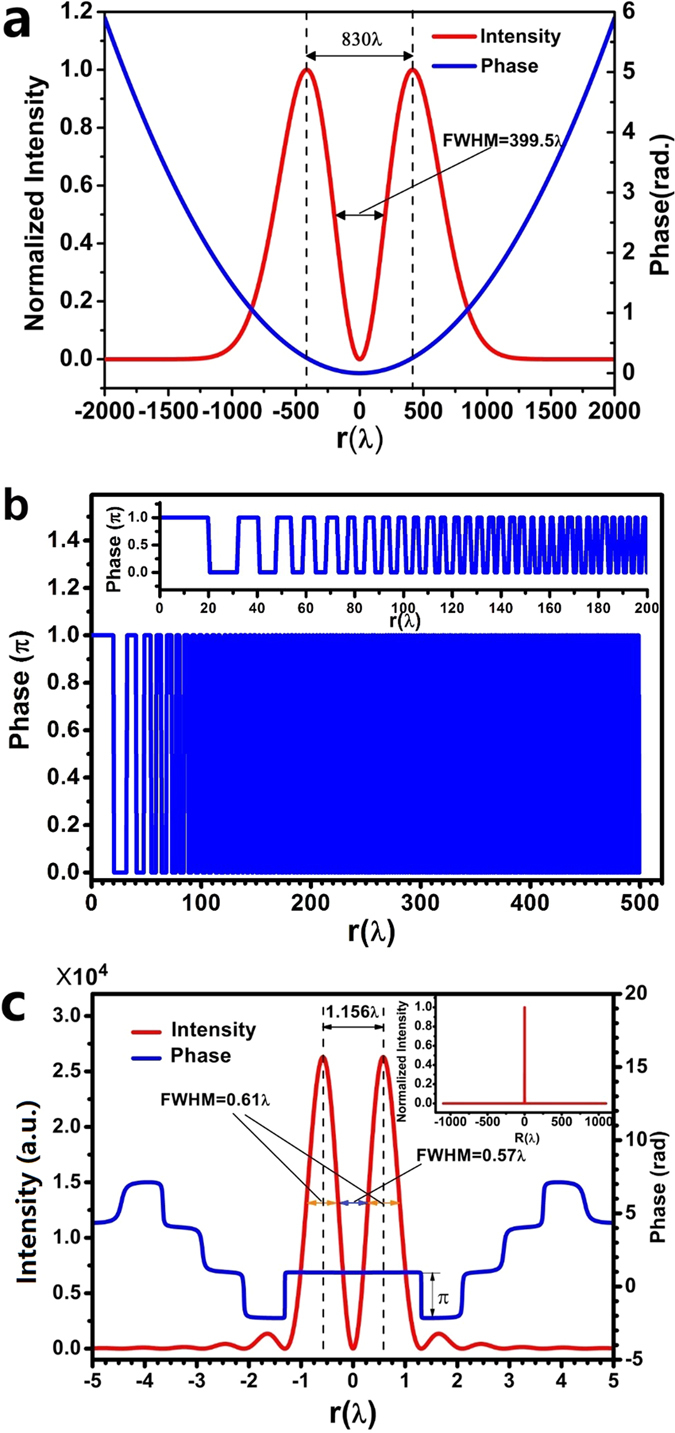
(**a**) The normalized theoretical intensity and phase distribution of the incident azimuthally polarized light; (**b**) the optimized lens phase distribution; (**c**) the designed intensity and phase distribution on the focal plane. The inset of (**b**) is the zoom-in plot of the lens phase distribution and the inset of (**c**) is the normalized intensity on the focal plane.

The incident beam intensity profile is Laguerre–Gaussian with *w*_0_ = 331 μm and *z* = 276 mm (Fig. 2(a)), and shows a peak-peak diameter of 830 λ and a hollow ring FWHM of 399.5 λ. According to the vectorial angular spectrum method, there is only an azimuthal component in the electrical field diffracted by the circularly symmetrical lens. The electrical field on the focal plane at z = z_*f*_ is given by Equation 2, which was obtained by applied the vectorial angular spectrum diffraction formulas^21^,^27^ to the azimuthally polarized light with circular symmetrical intensity distribution in cylindrical coordinate.


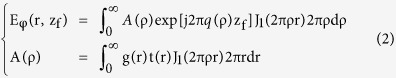


where *φ* denotes the polarization direction of the incident light, *r* and *ρ* are radial coordinates in the spatial and frequency domains, respectively, *g*(*r*) and *t*(*r*) are the incident beam electrical field distribution and the lens transmittance function, respectively, *J*_1_ is the first-order Bessel function, and *q*(*ρ*) = (1/λ^2^-*ρ*^2^)^1/2^.

The radius of the lens is *R* = 500 λ, its focal length is *f *= 600 λ, and its numerical aperture (NA) is *sin*[*arctan*( *f*/*R*)] = 0.64. Therefore, the corresponding diffraction limit is 0.78 λ (0.5 λ/NA) and the super-oscillation criterion is 0.59 λ (0.38 λ/NA)^40^. The value of *T* is 500 nm, which is smaller than the incident wavelength of 632.8 nm. Figure 2(b) gives the optimized phase spatial distribution on the lens in the area with radius less than 120 λ (the details of the phase spatial distribution can be found in the supplementary materials, where the phase 0 and π correspond to t_Si3N4_ values of 0 and 348 nm respectively). The theoretically optimized electrical field distribution on the focal plane was depicted with respect to the radial coordinate in Fig. 2(c), which shows the cross-section of the optical hollow ring intensity (red). The peak-peak diameter of the hollow ring is about 1.156 λ and the FWHM (as indicated by the blue arrows) is about 0.57 λ, which is almost half of the peak-peak diameter, and smaller than the diffraction limit of 0.78 λ (0.5 λ/NA) and the super-oscillation criterion of 0.59 λ. The sidelobe intensity on the focal plane was found to be less than 5.1% of the hollow ring peak intensity, leading to a very clear field of view in the region [−1050 λ, +1050 λ], as shown in the inset of Fig. 2(c). We also found that the FWHM of each peak (as indicated by the orange arrows) is about 0.61 λ, which is also smaller than the diffraction limit of 0.78 λ. This sub-diffraction feature was also reflected in the phase distribution (blue curve), which shows a sharp phase inversion, or a phase change of π, at the first minimum at *r* = 1.3 λ. This sharp phase change is a direct evidence of super-oscillation.

### Numerical simulations

**Figure 3:**
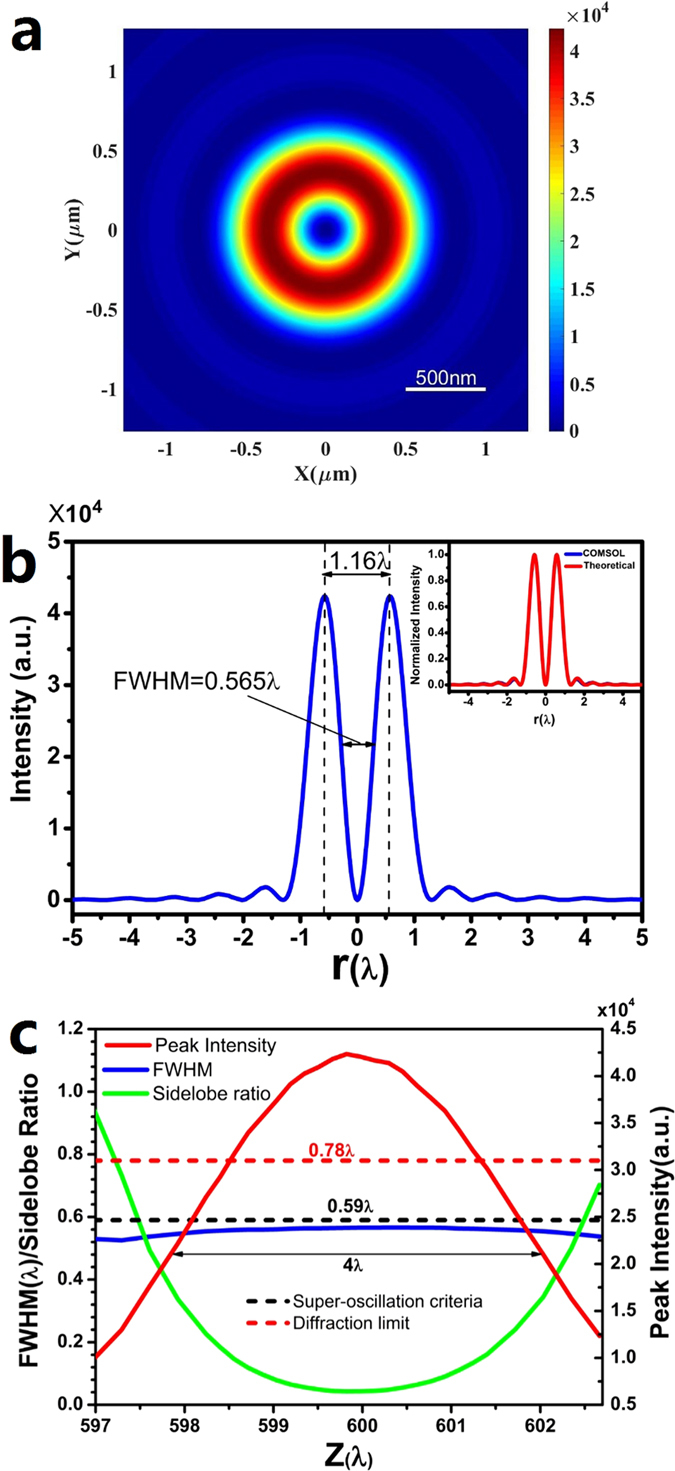
COMSOL Multiphysics simulation results. (**a**) The color map of the intensity distribution on the focal plane; (**b**) the variation of the optical intensity along the radial direction on the focal plane; (**c**) the change in the FWHM, peak intensity, and sidelobe ratio along the propagation axis Z. The inset of (**b**) gives the comparison between the theoretical design and the COMSOL simulation.

The COMSOL Multiphysics software was used to conduct the simulation of the optimized lens with real physical Si_3_N_4_ ring structures. Following the optimized phase distribution, a real lens structure, as given in Fig. 1(b), was constructed in the COMSOL Multiphysics (The detailed structure information can be found in Table 1 in the Supplementary Information). The ring thickness is *t* = 348 nm for phase π and a Si_3_N_4_ refractive index of 1.91. The parameters of the incident azimuthally polarized wave are the same as those used in the theoretical design. In the simulation, scattering boundary condition and perfectly matched layer was used to avoid unphysical reflection from the boundary. According to the simulation results, the focal length was 599.8 λ, which is quite close to the value of 600 λ in our theoretical design. Figure 3(a) gives the color map of the optical intensity on the focal plane. Figure 3(b) plots the corresponding optical intensity against the radial coordinate, which shows a hollow ring FWHM of 0.565 λ and a peak-peak diameter of 1.16 λ. Both the FWHM and the peak-peak diameter are quite close to their corresponding values from the theoretical design. In the inset of Fig. 3(b), the focal plane optical intensity distribution is plotted for both theoretical design and COMSOL simulation for comparison, and two plots show an excellent agreement. According to the COMSOL simulation, the variation of the FWHM, peak intensity, and sidelobe ratio (the ratio of the maximum sidelobe to the peak intensity) were plotted along the optical axis in Fig. 3(c). We found that the FWHM of the hollow ring was smaller than the super-oscillation criterion of 0.59 λ in the area of 597 λ < z< 602.7 λ around the focal point z = 599.8 λ. In the area of z between 599 λ and 600.8 λ, the sidelobe ratio was less than 10%. The FWHM of the hollow ring in the z direction (propagation direction) was about 4 λ, as indicated by the arrows in Fig. 3(c).

## Results and Discussion

### Lens fabrication

**Figure 4:**
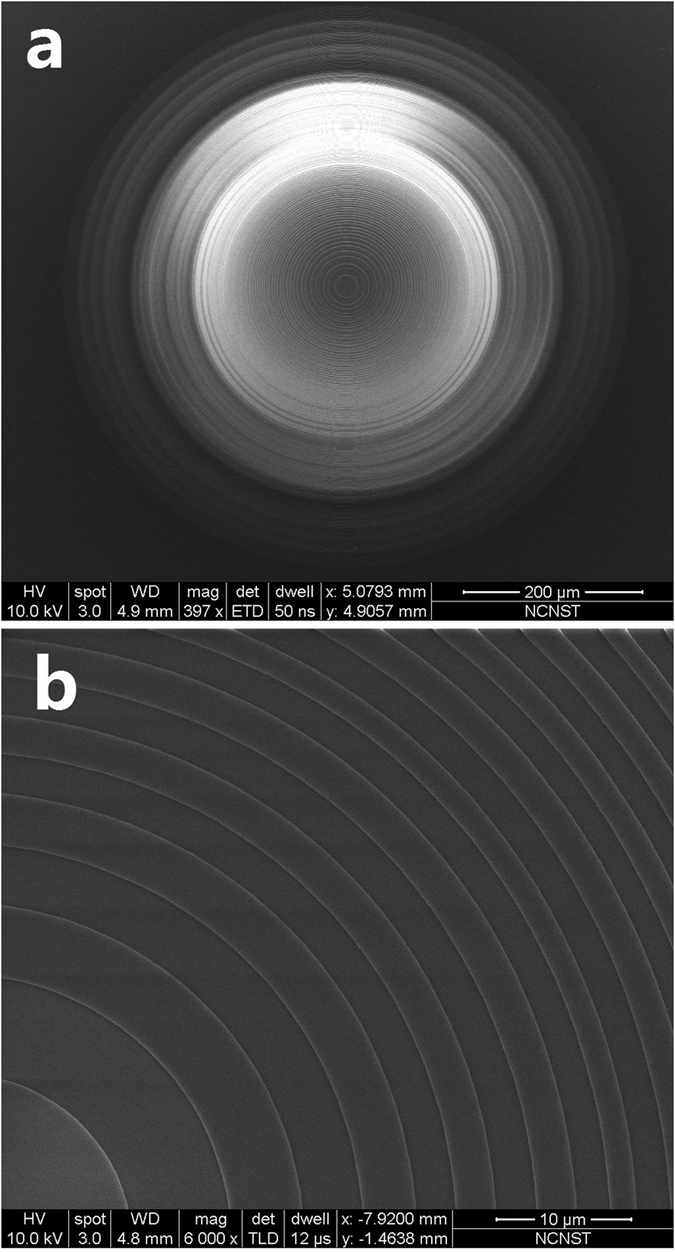
(**a**) The SEM images of the micro lens, and (**b**) the zoom-in of the lens central part.

A micro lens was fabricated using electron-beam lithography and dry etching. The detailed geometrical structure of the micro lens is shown in Table 1 in the Supplementary Information. A 500-μm thick sapphire glass was used as the lens substrate. A Si_3_N_4_ layer was first deposited on the substrate with PECVD coating, and its refractive index was characterized with ellipsometry, which yielded a refractive index of 1.91. The thickness of this dielectric layer was about 348 nm, corresponding to the relative phase change of π. Dry etching was adopted to form the Si_3_N_4_ dielectric ring structures. Figure 4(a,b) shows the SEM images of the micro lens.

### Experimental setup

**Figure 5:**
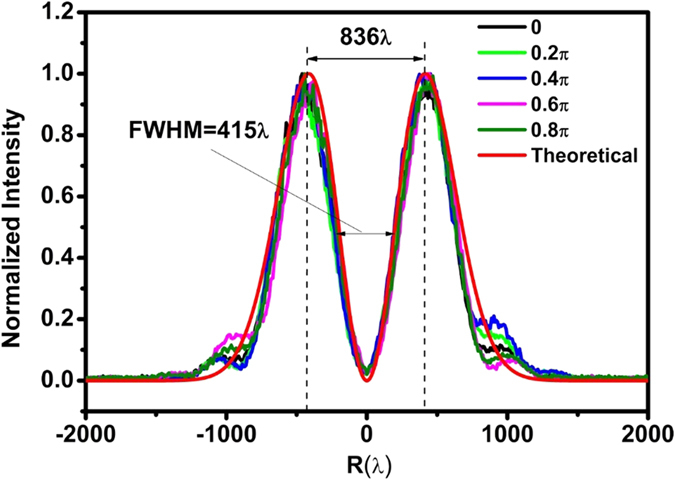
The intensity distribution of the incident azimuthally polarized wave at five different directions with angles of 0, 0.2 π, 0.4 π, 0.6 π, and 0.8 π crossing the beam’s center on the lens input surface.

To experimentally generate a sub-diffraction optical hollow ring, the incident azimuthally polarized wave was first produced by illuminating an s-wave plate (Workshop of Photonics, Lithuania) with a normal incident linearly polarized Gaussian beam at a wavelength of 632.8 nm from a He-Ne laser. The power of the azimuthally polarized light was about 4 mW. In Fig. 5, the intensity profile of the azimuthally polarized wave on the incident surface was illustrated for five different directions with angles of 0, 0.2 π, 0.4 π, 0.6 π, and 0.8 π crossing the beam’s center. The peak-peak diameter and FWHM of the incident azimuthally polarized wave were about 836 λ and 415 λ, respectively, similar to the theoretical values of 830 λ and 399.5 λ, respectively, in the design (Fig. 2(a)). We also noticed that the intensity distribution did not exhibit perfectly circular symmetry because of difficulties in optical alignment.

In the experiment, the azimuthally polarized wave was normally incident on the lens from the substrate side. The diffracted optical intensity distribution was measured after the lens with a tapered optical fiber probe (CFN-100 of Nanonics Imaging, Ltd., Israel) mounted on a 3-D piezo nanopositioner (P-561.3CD of Physik Instrumente GmbH & Co., Germany). The probe tip diameter was 100 nm. Although the probe size has convolution effect on the experimental result, it was found to be small and can be ignored in our case. The spatial resolution and scanning range of the nanopositioner were about 10 nm and 100 μm, respectively, for each of the x, y, and z axes. The collected photons were detected by a single photon detector (SPCM50A/M of Thorlabs, Inc., USA). By moving the 3-D nanopositioner, the fiber probe was able to scan the optical intensity distribution in the plane perpendicular to the optical axis at different distances.

### Experimental generation of sub-diffraction hollow ring

**Figure 6:**
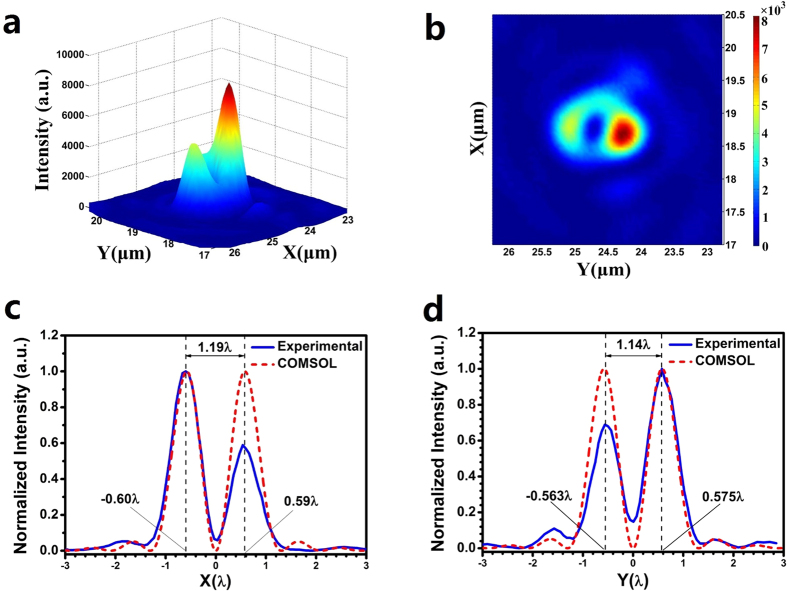
The (**a**) 3D and (**b**) 2D color maps of the measured optical intensity on the focal plane; the optical intensity distribution along the (**c**) x-axis and (**b**) y-axis.

The focal plane was found to be approximately 380 μm (600.5 λ), which was similar to the theoretically predicted value of 600 λ and the COMSOL simulation result of 599.8 λ. Figure 6(a,b) give the 3D and 2D color maps of the optical intensity measured on the focal plane, which show a clear hollow ring structure. Due to the difficulties in the optical alignment mentioned above, the focal hollow ring was not symmetrical. The optical intensity varied around the circumference of the central peak lobe. To evaluate the size of the hollow ring, the normalized intensity distribution was plotted along the x direction and y direction across the center of the hollow ring in Fig. 6(c,d), respectively. The peak-peak diameter of the hollow ring was 1.19 λ and 1.14 λ in the x direction and y direction, respectively, and these values are quite close to the value of 1.16 λ obtained in the COMSOL simulation, depicted in Fig. 3(b). For further comparison, the normalized COMSOL simulation result is also given in the Fig. 6(c,d).

**Figure 7:**
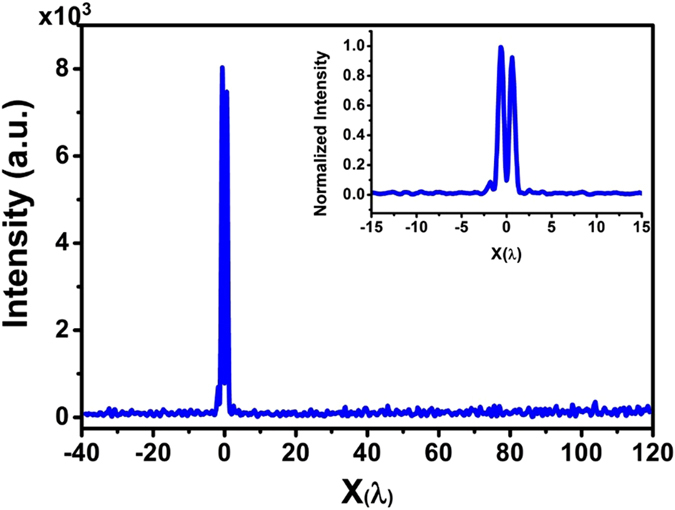
The optical intensity distribution taken in a large range in the x-direction across the focus center on the focal plane.

Except for the symmetry, the experimental results show a good agreement with the intensity shape from the theoretical simulation. The hollow ring FWHM is 444 nm (0.702 λ) and 356 nm (0.563 λ) in the x direction and y direction, respectively, and both are smaller than the diffraction limit of 0.78 λ. The FWHM in the y direction is even smaller than the super-oscillation criterion of 0.59 λ. To better evaluate the hollow ring’s size, the FWHM was measured along the 16 different directions with equal angle steps of 24°. We obtained an average FWHM of 386 nm (0.61 λ), which was smaller than the diffraction limit of 0.78 λ and close to the super-oscillation criterion of 0.59 λ. The corresponding average peak-peak diameter was about 720 nm (1.14 λ), which is also similar to the COMSOL simulation result of 1.16 λ, as shown in Fig. 3(b). A long-range scan was taken along the x direction to investigate a large area of optical intensity on the focal plane, as shown in Fig. 7, which gives a clear field of view in the area of [−39 λ, 120 λ]. The measured maximum sidelobe was less than 10% of the maximum peak intensity. Numerical investigation was conducted to find out the influence of fabrication error and optical misalignment on the optical intensity distribution on the focal plane. It was found that off-axis incident and tilted incident have major contributions to the asymmetrical optical intensity distribution on the focal plane.

## Conclusions

It has been demonstrated that a sub-diffraction hollow ring structure can be obtained in the far-field by shaping an azimuthally polarized wave with a micro planar lens based on the binary phase mask. The planar lens was designed and fabricated with an ultra-long focal length of 600 λ and small NA of 0.64. Experimental results showed that a hollow ring was generated with an FWHM of 0.61 λ, which is smaller than the diffraction limit of 0.78 λ. The measured peak-peak diameter of the hollow ring was about 1.14 λ and the sidelobe’s ring intensity was less than 10% of the central lobe’s maximum intensity. Compared with conventional lenses, sub-diffraction lenses can achieve the same FWHM with smaller NA, and therefore realize a much longer focal length and working distance, which is important for detecting the information deep inside the sample. A hollow ring with FWHM smaller than 0.36 λ (or even smaller) is also expected to be achieved by further increasing the NA value and employing more phase values during the design and fabrication stages. Such a sub-diffraction planar lens with an ultra-long focal length has great potential in further improving the optical resolution of stimulated emission depletion (STED) microscopy, optical tweezers, and nanolithography. This planar lens is also attractive due to its unique properties, including its small size, light weight, and ability to be easily integrated. This method can also be adopted for the generation of sub-diffraction hollow ring in other spectrum ranges, such as infrared and terahertz.

## Additional Information

**How to cite this article**: Chen, G. *et al*. Generation of a sub-diffraction hollow ring by shaping an azimuthally polarized wave. *Sci. Rep.* **6**, 37776; doi: 10.1038/srep37776 (2016).

**Publisher's note:** Springer Nature remains neutral with regard to jurisdictional claims in published maps and institutional affiliations.

## Supplementary Material

Supplementary Materials

## References

[b1] Zhan Q. Cylindrical vector beams: from mathematical concepts to applications. Adv. Opt. Photon. 1, 1–57 (2009).

[b2] Hell S. W. Far-field optical nanoscopy. Science 316, 1153–1158 (2007). 17525330 10.1126/science.1137395

[b3] Zhang D. W. & Yuan X. C. Optical doughnut for optical tweezers. Opt. Lett 28, 740–742 (2003). 12747724 10.1364/ol.28.000740

[b4] Gan Z., Cao Y., Evans R. A. & Gu M. Three-dimensional deep sub-diffraction optical beam lithography with 9 nm feature size. Nat. Comm 4, 1–7 (2013). 10.1038/ncomms3061 23784312

[b5] Kuang C., Liu Y., Hao X., Luo D. & Liu X. Creating attoliter detection volume by microsphere photonic nanojet and fluorescence depletion. Opt. Commun 285, 402–406 (2012).

[b6] Hao X. A., Kuang C. F., Wang T. T. & Liu X. Phase encoding for sharper focus of the azimuthally polarized beam. Opt. Lett 35, 3928–3930 (2010). 21124568 10.1364/OL.35.003928

[b7] Youngworth K. & Brown T. Focusing of high numerical aperture cylindrical-vector beams. Opt. Exp. 7, 77–87 (2000). 10.1364/oe.7.000077 19404372

[b8] Helseth L. E. Smallest focal hole. Opt. Commun. 257, 1–8 (2006).

[b9] Zhan Q. & Leger J. Focus shaping using cylindrical vector beams. Opt. Exp 10, 324–331 (2002). 10.1364/oe.10.000324 19436363

[b10] Yuan G. H., Wei S. B. & Yuan X. C. Nondiffracting transversally polarized beam. Opt. Lett 36, 3479–3481 (2011). 21886250 10.1364/OL.36.003479

[b11] Tian B. & Pu J. Tight focusing of a double-ring-shaped, azimuthally polarized beam. Opt. Lett 36, 2014–2016 (2011). 21633433 10.1364/OL.36.002014

[b12] Lalithambigai K. . Generation of subwavelength super-long dark channel using high NA lens axicon. Opt. Lett 37, 999–1001 (2012). 22446203 10.1364/OL.37.000999

[b13] Hao X., Kuang C., Wang T. & Liu X. Manipulation of doughnut focal spot by image inverting interferometry. Opt. Lett 37, 821–823 (2012). 22378405 10.1364/OL.37.000821

[b14] Chen W. . Large scale manipulation of the dark spot by phase modulation of azimuthally polarized light. Opt. Commun 349, 125–131 (2015).

[b15] Luo J. . Tight focusing of radially and azimuthally polarized light with plasmonic metalens. Opt. Commun 356, 445–450 (2015).

[b16] Pendry J. B. Negative refraction makes a perfect lens. Phys. Rev. Lett. 85, 3966 (2000). 11041972 10.1103/PhysRevLett.85.3966

[b17] Fang N., Lee H., Sun C. & Zhang X. Sub-diffraction-limited optical imaging with a silver superlens. Science 308, 534–537 (2005). 15845849 10.1126/science.1108759

[b18] Luo J. . Fabrication of anisotropically arrayed nano-slots metasurfaces using reflective plasmonic lithography. Nano Scale 7, 18805 (2015). 10.1039/c5nr05153c 26507847

[b19] Gao P. . Enhancing aspect profile of half-pitch 32 nm and 22 nm lithography with plasmonic cavity lens. Appl. Phys. Lett. 106, 093110 (2015).

[b20] Zhao Z. . Going far beyond the near-field diffraction limit via plasmonic cavity lens with high spatial frequency spectrum off-axis illumination. Sci. Rep. 5, 15320 (2015). 26477856 10.1038/srep15320 PMC4609954

[b21] Kotlyar V. V. . Analysis of the shape of a subwavelength focal spot for the linearly polarized light. Appl. Opt 52, 330–339 (2013). 23338178 10.1364/AO.52.000330

[b22] Yuan G. . Planar super-oscillatory lens for sub-diffraction optical needles at violet wavelengths. Sci. Rep. 4, 6333 (2014). 25208611 10.1038/srep06333 PMC4160710

[b23] Rogers E. T. F. . A super-oscillatory lens optical microscope for subwavelength imaging. Nat. Mater 11, 432 (2012). 22447113 10.1038/nmat3280

[b24] Rogers E. T. F. & Zheludev N. I. Optical super-oscillations: sub-wavelength light focusing and super-resolution imaging. J. Opt 15, 094008 (2013).

[b25] Yuan G., Rogers E. T. F., Roy T., Shen Z. & Zheludev N. I. Flat super-oscillatory lens for heat-assisted magnetic recording with sub-50 nm resolution. Opt. Exp. 22, 6428–6437 (2014). 10.1364/OE.22.006428 24663991

[b26] Qin F. . Shaping a subwavelength needle with ultra-long focal length by focusing azimuthally polarized light. Sci. Rep. 5, 09977 (2015). 10.1038/srep09977 PMC4421869 25943500

[b27] Liu T., Tan J. B., Liu J. & Wang H. T. Vectorial design of super-oscillatory lens. Opt. Exp. 21, 15090–15101 (2013). 10.1364/OE.21.015090 23842296

[b28] Wen Z. Q., He Y. H., Li Y. Y., Chen L. & Chen G. Super-oscillation focusing lens based on continuous amplitude and binary phase modulation. Opt. Exp. 22, 22163–22171 (2014). 10.1364/OE.22.022163 25321591

[b29] Chen G. . Super-oscillation far-field focusing lens based on ultra-thin width-varied metallic slit array. IEEE Photon. Technol. Lett. 28, 335–338 (2016).

[b30] Chen G. . Far-field sub-diffraction focusing lens based on binary amplitude-phase mask for linearlypolarized light. Opt. Exp. 24, 11002–11008 (2016). 10.1364/OE.24.011002 27409922

[b31] Chen G. . Super-oscillatory focusing of circularly polarized light by ultra-long focal Length planar lens based on binary amplitude-phase modulation, Sci. Rep. 6, 29068 (2016). 27353239 10.1038/srep29068 PMC4926254

[b32] Smith D. R., Pendry J. B. & Wiltshire M. C. K. Metamaterials and negative refractive index. Science 305, 788–792 (2004). 15297655 10.1126/science.1096796

[b33] Shen Y., Ko H. Y., Ai Q., Peng S. M. & Jin B. Y. Molecular split-ring resonators based on metal string complexes. J. Phys. Chem. C 118, 3766 (2014).

[b34] Tang D. . Ultrabroadband superoscillatory lens composed by plasmonic metasurfaces for subdiffraction light. Laser and Photonics Reviews 9, 713–719 (2015).

[b35] Huang F. M. & Zheludev N. I. Super-resolution without evanescent waves. Nano Lett. 9, 1249–1254 (2009). 19182908 10.1021/nl9002014

[b36] Wong A. M. H. & Eleftheriades G. V. An optical super-microscope for far-field, real-time imaging beyond the diffraction limit. Sci. Rep. 3, 1715 (2013). 23612684 10.1038/srep01715 PMC3634104

[b37] Wang C. . Super-resolution optical telescopes with local light diffraction shrinkage. Sci. Rep. 5, 18485 (2015). 26677820 10.1038/srep18485 PMC4683440

[b38] Jin N. & Rahmat-Samii Y. Advances in particle swarm optimization for antenna designs: real-number, binary, single-objective and multi-objective implementations. IEEE Trans. Antenn. Propag. 55, 556–567 (2007).

[b39] Nesterov A. V. & V. G Niziev. Laser beams with axially symmetric polarization. J. Phys. D Appl. Phys. 33, 15 (2000).

[b40] Huang K. . Optimization-free superoscillatory lens using phase and amplitude masks. Laser Photon. Rev. 8, 152–157 (2014).

