# Supplementary material for: Generation of a sub-diffraction hollow ring by shaping an azimuthally polarized wave
Source: Sci Rep. 2016 Nov 23;6:37776. doi: 10.1038/srep37776 (PMC5120339; doi:10.1038/srep37776)
Supplement: Supplementary Materials [file srep37776-s1.pdf]

Supporting Information

Generation of a sub-diffraction hollow ring by shaping an azimuthally polarized wave

Gang Chen[1, †], Zhi-xiang Wu[1], An-ping Yu[1], Zhi-hai Zhang[1], Zhong-quan Wen[1, ††], Kun Zhang[1,2], Lu-ru Dai [2, †††], Sen-lin Jiang[1], Yu-yan Li[1], Li Chen[1], Chang-tao Wang[3], and Xian-gang Luo[3]

[†, ††, †††]Corresponding Author: E-mail: gchen1@cqu.edu.cn, wenzq@cqu.edu.cn, dai@nanoctr.cn

[1]Key Laboratory of Optoelectronic Technology and Systems (Chongqing University), Ministry of Education, and Key Disciplines Lab of Novel Micro-nano Devices and System Technology, Chongqing University,173 Shazheng Street, Shapingba, Chongqing 400044, China.
[2]National Center for Nanoscience and Technology, No.11 Zhong Guan CunBei Yi Tiao, Beijing 100190, China.
[3]State Key Laboratory of Optical Technologies on Nano-Fabrication and Micro-Engineering, Institute of Optics and Electronics, Chinese Academy of Science, P. R. Box 350, Chengdu 610209, China

Supplementary

Table S1. the geometry of the micro-lens (T=500 nm)

i[a)]	Ri [nm] [b)]	t_{Si3N4} [nm] [c)]	i	Ri [nm]	t_{Si3N4} [nm]	i	Ri [nm]	t_{Si3N4} [nm]	i	Ri [nm]	t_{Si3N4} [nm]
0	0	348	48	24000	348	96	48000	0	144	72000	0
1	500	348	49	24500	348	97	48500	0	145	72500	0
2	1000	348	50	25000	348	98	49000	348	146	73000	348
3	1500	348	51	25500	348	99	49500	348	147	73500	348
4	2000	348	52	26000	0	100	50000	348	148	74000	348
5	2500	348	53	26500	0	101	50500	348	149	74500	0
6	3000	348	54	27000	0	102	51000	0	150	75000	0
7	3500	348	55	27500	0	103	51500	0	151	75500	0
8	4000	348	56	28000	0	104	52000	0	152	76000	0
9	4500	348	57	28500	0	105	52500	0	153	76500	348
10	5000	348	58	29000	0	106	53000	0	154	77000	348
11	5500	348	59	29500	0	107	53500	348	155	77500	348
12	6000	348	60	30000	0	108	54000	348	156	78000	0
13	6500	348	61	30500	348	109	54500	348	157	78500	0
14	7000	348	62	31000	348	110	55000	348	158	79000	0
15	7500	348	63	31500	348	111	55500	348	159	79500	348
16	8000	348	64	32000	348	112	56000	0	160	80000	348
17	8500	348	65	32500	348	113	56500	0	161	80500	348
18	9000	348	66	33000	348	114	57000	0	162	81000	0
19	9500	348	67	33500	348	115	57500	0	163	81500	0
20	10000	348	68	34000	348	116	58000	348	164	82000	0
21	10500	348	69	34500	0	117	58500	348	165	82500	348
22	11000	348	70	35000	0	118	59000	348	166	83000	348
23	11500	348	71	35500	0	119	59500	348	167	83500	348
24	12000	348	72	36000	0	120	60000	0	168	84000	0
25	12500	348	73	36500	0	121	60500	0	169	84500	0
26	13000	0	74	37000	0	122	61000	0	170	85000	0
27	13500	0	75	37500	348	123	61500	0	171	85500	348
28	14000	0	76	38000	348	124	62000	348	172	86000	348
29	14500	0	77	38500	348	125	62500	348	173	86500	348
30	15000	0	78	39000	348	126	63000	348	174	87000	0
31	15500	0	79	39500	348	127	63500	348	175	87500	0
32	16000	0	80	40000	348	128	64000	0	176	88000	348
33	16500	0	81	40500	0	129	64500	0	177	88500	348
34	17000	0	82	41000	0	130	65000	0	178	89000	348
35	17500	0	83	41500	0	131	65500	0	179	89500	0
36	18000	0	84	42000	0	132	66000	348	180	90000	0
37	18500	0	85	42500	0	133	66500	348	181	90500	0
38	19000	0	86	43000	0	134	67000	348	182	91000	348
39	19500	0	87	43500	348	135	67500	0	183	91500	348
40	20000	0	88	44000	348	136	68000	0	184	92000	348
41	20500	348	89	44500	348	137	68500	0	185	92500	0
42	21000	348	90	45000	348	138	69000	0	186	93000	0
43	21500	348	91	45500	348	139	69500	348	187	93500	348
44	22000	348	92	46000	348	140	70000	348	188	94000	348
45	22500	348	93	46500	0	141	70500	348	189	94500	348
46	23000	348	94	47000	0	142	71000	348	190	95000	0
47	23500	348	95	47500	0	143	71500	0	191	95500	0

i	Ri [nm]	t_{Si3N4} [nm]	i	Ri [nm]	t_{Si3N4} [nm]	i	Ri [nm]	t_{Si3N4} [nm]	i	Ri [nm]	t_{Si3N4} [nm]
192	96000	0	240	120000	348	288	144000	0	336	168000	348
193	96500	348	241	120500	0	289	144500	348	337	168500	0
194	97000	348	242	121000	0	290	145000	348	338	169000	0
195	97500	0	243	121500	348	291	145500	0	339	169500	348
196	98000	0	244	122000	348	292	146000	0	340	170000	348
197	98500	0	245	122500	0	293	146500	348	341	170500	0
198	99000	348	246	123000	0	294	147000	348	342	171000	348
199	99500	348	247	123500	348	295	147500	0	343	171500	348
200	100000	0	248	124000	348	296	148000	348	344	172000	0
201	100500	0	249	124500	0	297	148500	348	345	172500	348
202	101000	0	250	125000	0	298	149000	0	346	173000	348
203	101500	348	251	125500	348	299	149500	0	347	173500	0
204	102000	348	252	126000	348	300	150000	348	348	174000	348
205	102500	0	253	126500	0	301	150500	0	349	174500	348
206	103000	0	254	127000	0	302	151000	0	350	175000	0
207	103500	0	255	127500	348	303	151500	348	351	175500	348
208	104000	348	256	128000	348	304	152000	348	352	176000	348
209	104500	348	257	128500	0	305	152500	0	353	176500	0
210	105000	0	258	129000	0	306	153000	0	354	177000	348
211	105500	0	259	129500	348	307	153500	348	355	177500	348
212	106000	348	260	130000	348	308	154000	0	356	178000	0
213	106500	348	261	130500	0	309	154500	0	357	178500	348
214	107000	348	262	131000	0	310	155000	348	358	179000	348
215	107500	0	263	131500	348	311	155500	348	359	179500	0
216	108000	0	264	132000	348	312	156000	0	360	180000	348
217	108500	348	265	132500	0	313	156500	348	361	180500	348
218	109000	348	266	133000	0	314	157000	348	362	181000	0
219	109500	0	267	133500	348	315	157500	0	363	181500	348
220	110000	0	268	134000	348	316	158000	0	364	182000	348
221	110500	0	269	134500	0	317	158500	348	365	182500	0
222	111000	348	270	135000	0	318	159000	0	366	183000	348
223	111500	348	271	135500	348	319	159500	0	367	183500	348
224	112000	0	272	136000	348	320	160000	348	368	184000	0
225	112500	0	273	136500	0	321	160500	0	369	184500	348
226	113000	348	274	137000	0	322	161000	0	370	185000	0
227	113500	348	275	137500	348	323	161500	348	371	185500	0
228	114000	0	276	138000	348	324	162000	348	372	186000	348
229	114500	0	277	138500	0	325	162500	0	373	186500	0
230	115000	348	278	139000	348	326	163000	348	374	187000	0
231	115500	348	279	139500	348	327	163500	348	375	187500	348
232	116000	348	280	140000	0	328	164000	0	376	188000	0
233	116500	0	281	140500	0	329	164500	0	377	188500	0
234	117000	0	282	141000	348	330	165000	348	378	189000	348
235	117500	348	283	141500	348	331	165500	0	379	189500	0
236	118000	348	284	142000	0	332	166000	0	380	190000	348
237	118500	0	285	142500	0	333	166500	348	381	190500	348
238	119000	0	286	143000	348	334	167000	0	382	191000	0
239	119500	348	287	143500	0	335	167500	0	383	191500	348

i	Ri [nm]	t_{Si3N4} [nm]	i	Ri [nm]	t_{Si3N4} [nm]	i	Ri [nm]	t_{Si3N4} [nm]	i	Ri [nm]	t_{Si3N4} [nm]
384	192000	348	432	216000	348	480	240000	0	528	264000	0
385	192500	0	433	216500	0	481	240500	348	529	264500	348
386	193000	348	434	217000	348	482	241000	0	530	265000	0
387	193500	0	435	217500	0	483	241500	348	531	265500	348
388	194000	0	436	218000	0	484	242000	0	532	266000	0
389	194500	348	437	218500	348	485	242500	348	533	266500	348
390	195000	0	438	219000	0	486	243000	348	534	267000	0
391	195500	0	439	219500	348	487	243500	0	535	267500	348
392	196000	348	440	220000	0	488	244000	348	536	268000	0
393	196500	0	441	220500	0	489	244500	0	537	268500	0
394	197000	348	442	221000	348	490	245000	348	538	269000	348
395	197500	348	443	221500	0	491	245500	0	539	269500	0
396	198000	0	444	222000	348	492	246000	348	540	270000	348
397	198500	348	445	222500	0	493	246500	348	541	270500	0
398	199000	0	446	223000	0	494	247000	0	542	271000	348
399	199500	0	447	223500	348	495	247500	348	543	271500	0
400	200000	348	448	224000	0	496	248000	0	544	272000	348
401	200500	0	449	224500	348	497	248500	348	545	272500	0
402	201000	0	450	225000	0	498	249000	0	546	273000	348
403	201500	348	451	225500	0	499	249500	348	547	273500	0
404	202000	0	452	226000	348	500	250000	348	548	274000	348
405	202500	348	453	226500	0	501	250500	0	549	274500	348
406	203000	348	454	227000	348	502	251000	348	550	275000	0
407	203500	0	455	227500	0	503	251500	0	551	275500	348
408	204000	348	456	228000	0	504	252000	348	552	276000	0
409	204500	0	457	228500	348	505	252500	0	553	276500	348
410	205000	0	458	229000	0	506	253000	348	554	277000	0
411	205500	348	459	229500	348	507	253500	0	555	277500	348
412	206000	0	460	230000	0	508	254000	0	556	278000	0
413	206500	348	461	230500	348	509	254500	348	557	278500	348
414	207000	348	462	231000	348	510	255000	0	558	279000	0
415	207500	0	463	231500	0	511	255500	348	559	279500	348
416	208000	348	464	232000	348	512	256000	0	560	280000	0
417	208500	0	465	232500	0	513	256500	348	561	280500	348
418	209000	0	466	233000	348	514	257000	0	562	281000	0
419	209500	348	467	233500	348	515	257500	348	563	281500	348
420	210000	0	468	234000	0	516	258000	0	564	282000	348
421	210500	348	469	234500	348	517	258500	0	565	282500	0
422	211000	0	470	235000	0	518	259000	348	566	283000	348
423	211500	0	471	235500	348	519	259500	0	567	283500	0
424	212000	348	472	236000	0	520	260000	348	568	284000	348
425	212500	0	473	236500	0	521	260500	0	569	284500	0
426	213000	348	474	237000	348	522	261000	348	570	285000	348
427	213500	348	475	237500	0	523	261500	0	571	285500	0
428	214000	0	476	238000	348	524	262000	348	572	286000	348
429	214500	348	477	238500	0	525	262500	0	573	286500	0
430	215000	0	478	239000	348	526	263000	0	574	287000	348
431	215500	348	479	239500	0	527	263500	348	575	287500	0

i	Ri [nm]	t_{Si3N4} [nm]	i	Ri [nm]	t_{Si3N4} [nm]	i	Ri [nm]	t_{Si3N4} [nm]	i	Ri [nm]	t_{Si3N4} [nm]
576	288000	348	591	295500	348	606	303000	0	621	310500	348
577	288500	0	592	296000	0	607	303500	348	622	311000	0
578	289000	348	593	296500	348	608	304000	0	623	311500	348
579	289500	0	594	297000	0	609	304500	348	624	312000	0
580	290000	348	595	297500	348	610	305000	0	625	312500	348
581	290500	0	596	298000	0	611	305500	348	626	313000	0
582	291000	348	597	298500	348	612	306000	0	627	313500	348
583	291500	0	598	299000	0	613	306500	348	628	314000	0
584	292000	348	599	299500	348	614	307000	0	629	314500	348
585	292500	348	600	300000	0	615	307500	348	630	315000	0
586	293000	0	601	300500	348	616	308000	0	631	315500	348
587	293500	348	602	301000	0	617	308500	348	632	316000	0
588	294000	0	603	301500	348	618	309000	0			
589	294500	348	604	302000	0	619	309500	348			
590	295000	0	605	302500	348	620	310000	0			

[a]The number of the ring; [b] the radius of the ring; [c] the thickness of the Si_3N_4 ring
